# The characteristics of microwave‐treated insoluble and soluble dietary fibers from grape and their effects on bread quality

**DOI:** 10.1002/fsn3.3705

**Published:** 2023-09-21

**Authors:** Duygu Baskaya‐Sezer

**Affiliations:** ^1^ Amasya Social Sciences Vocational School Amasya University Amasya Turkey

**Keywords:** fiber, polysaccharide, pomace, residue, waste

## Abstract

This study investigated the morphological and hydration properties of untreated and microwave (MW)‐treated isolate forms of soluble (SDF) and insoluble dietary fibers (IDF) obtained from grapes. Then, the rheological, textural, and other physical effects of the fibers (5% flour basis) were evaluated on bread quality. For this purpose, grape pomace was valorized as the juice extraction waste. MW significantly improved hydration properties of SDF and IDF by modifying their microstructures (*p* < .05). SDF had a clean‐cut morphology whereas IDF had an indented microstructure with a wrinkled surface. After MW treatment, deep grooves and holes were observed. These variations in the IDF structure were more extensive. DF additions influenced water absorption, mixing tolerance index, dough development time, dough stability, resistance to extension, extensibility, energy of the dough and hardness, cohesiveness, springiness, chewiness, weight loss, specific volume, crust color difference of the bread in comparison with the properties of control samples significantly (*p* < .05). IDF had especially pronounced effects on the dough and bread characteristics. SDF enrichment provided more comparable results with the control bread than IDF. The originality of this work is to characterize isolated (100% purity) SDFs and IDFs, then discuss their effects on semi (dough) and final (bread) product quality.

## Graphical abstract

One hundred percent pure insoluble and soluble dietary fibers were obtained from grape waste by microwave treatment. The fiber properties were examined. These fibers were used separately for bread enrichment and the quality characteristics of the bread were determined. The best result was found in bread with microwave‐treated soluble dietary fibers.

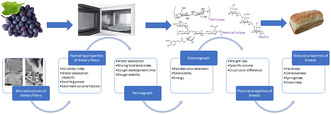

## 1 INTRODUCTION

Bread is a staple food typically prepared with medium‐hard wheat flour, salt, water, and yeast. White flour is the main ingredient; thus, it affects the dough and bread's properties substantially. However, high consumption of white flour can lead to the development of insulin resistance, type II diabetes, and other disorders triggered by diabetes due to white flour's high glycemic index. Dietary fiber (DF) is a good alternative that reduces or eliminates the negative effects of white flour on health.

In recent years, the role of DF in the prevention of diseases has been investigated. Ma et al. (2008) reported that DF intake is inversely associated with markers of systemic inflammation (a major cause of type II diabetes, atherosclerosis, and some cancers). As asserted by Arayici et al. (2022) and Nucci et al. (2021), insoluble DF (IDF) and soluble DF (SDF) intake is a protective factor in colon cancer. In addition, Kokubo et al. (2011) found an association between IDF consumption and stroke risk. Khan et al. (2018) stated that SDF lowers blood pressure and the risk of cardiovascular disease. For this purpose, The American Heart Association recommends a daily DF intake of 25–30 g from food, not supplement, but it has been reported that only 15 g are taken (Ucsfhealth, 2023).

Annual worldwide grape production was 78 million tons in 2020 (Faostat, 2020). However, 20%–30% of this crop by weight is wasted during processing as a by‐product: grape pomace (GP), which is primarily composed of skins, seeds, stems, and pulp. GP contains various antioxidant compounds (melatonin, anthocyanins, hydroxycinnamic acids, etc.). Unfortunately, this valuable portion constitutes an economic and ecological issue in terms of waste management. Efficient ways to reuse GP must be investigated to realize the excellent financial potential of this byproduct in the industry.

GP contains around 65% DF and more than 90% of the content is IDF (Bender et al., 2020). DF offers numerous benefits for human health, but it might deteriorate properties relevant to the processing and quality of some food products. For this purpose, it is necessary to modify the functional properties of SDFs and IDFs for the food product they will be added to by using effective methods.

Microwaves (MWs) have rapidly growing applications in food processing. MWs can alter the particle size, porosity, morphology, surface characteristics, hydration properties, molecular weight, thermal stability, crystallinity, monosaccharide composition, emulsion properties, and extraction yields of DFs (Gan et al., 2020; Jiang et al., 2020).

Regarding Antonić et al. (2020); Baldán et al. (2023); Bender et al. (2017); Boff et al. (2022); Curutchet et al. (2021); Nakov et al. (2020); Quiles et al. (2018) studies on this topic, the powder or flour forms of fruit and vegetable DFs were incorporated into products, and the final product characteristics were investigated. However, the findings of these studies were inevitably affected by the other compounds (sugar, protein, starch, and phenolic compounds) bound to these DF structures. On the other hand, the studies conducted on isolate forms of SDF and IDF extracted from the same sources are limited. To clearly reveal the effects of DFs on the product they are added to, the characteristics of their isolated forms should be investigated. This also gives rise to the possibility of discussing the effectiveness of the processing method only based on its effects on DF. By considering the importance of DF, this study aimed first to determine the functional and technological properties of untreated and MW‐treated isolated DFs and then to compare SDFs and IDFs regarding these properties. Its second aim was to investigate the effects of the obtained SDF and IDF on the rheological, physical, and textural properties of bread by using only a certain amount of flour.

## 2 MATERIALS AND METHODS

### 2.1 Preparation of raw material

GP was procured from Fruit Juice Company. After removing the stems, GP was frozen at −80°C in a deep freezer (ETS VF series) and then dried in a freeze dryer (Christ, Alpha 1–4 LSC plus) under 0.1 mPa at −55°C for 48 h to a moisture content of 5.0 ± 0.4%. For preparation of the untreated samples, 100 g of the dried sample was ground for 30 s in a grinder (Model no. 23120–56, Russell Hobbs). The ground samples were sieved through a 200‐mesh sieve (74 μm) (Endecotts). This sample was labeled as untreated GP. For preparation of the MW‐treated samples, 50 g of untreated GP was dispersed with deionized water (250 mL) in a beaker and treated in a microwave oven (HF15M561, Siemens) at the parameters (800 watt/120 s) determined by preliminary experiments. For drying step, thoroughly mixed MW‐treated GP was poured into petri dishes and placed in a −80°C freezer with cooling to 20°C within 30 min. Samples frozen for 48 h were immediately placed in a freeze dryer and dried at −55°C for 72 h to a moisture content of 4.7%–0.5%. By following these steps, untreated and MW‐treated GPs were produced in an amount (300 g each) sufficient for analyses and enriched breads. Afterward, the SDFs and IDFs of the dried samples were isolated. SDF samples consisted of 99.67 ± 0.19% SDF and IDF comprised 99.55 ± 0.08% of IDF samples.

### 2.2 Methods

#### 2.2.1 Isolation of SDF and IDF

The SDF and IDF of the untreated and the MW‐treated GP were isolated and TDF, SDF, and IDF contents of the samples were determined by following the AOAC 991.43 procedure (Lee et al., 1992). The SDF and IDF isolates were denoted as U‐SDF and U‐IDF for the untreated SDF and IDF, respectively; as MW‐SDF and MW‐IDF for the MW‐treated SDF and IDF, respectively. The samples of isolated IDF and SDF were stored at 4°C in sealed bottle until further analysis.

#### 2.2.2 Preparation of bread

Control bread was prepared using 100% (150 g) all‐purpose wheat flour (26.5% wet gluten, 14% moisture content, and 0.6% ash content – Gold Medal), 65% tap water, 3% compressed yeast (Saf), and 1.5% salt (Saxa). For the preparation of DF‐enriched bread samples, 5% DF fractions (U‐SDF, U‐IDF, MW‐SDF, or MW‐IDF) were added to the dough prepared with 95% wheat flour. Since only the effect of SDF and IDF on dough and bread properties was investigated in the study, the amount of water was kept constant in all formulations.

While preparing the dough samples, all ingredients were added and mixed in a Kitchen Aid mixer (5KSM125) for 10 min. The doughs were fermented in a proofer (Wachtel GmbH & Co.) at 35°C and 85% humidity for 70 min. Then, 250 g of dough was weighed into 20 cm × 10 cm × 7 cm pans and placed for 60 min in the proofer one more time. Dough fermentation parameters were used in proofing. In each run, three pieces of the dough were baked at 220°C for 25 min in a convection oven (Wachtel GmbH & Co.). After baking, all bread loaves were cooled for 60 min at room temperature. Then, the breads were subjected to measurements.

#### 2.2.3 Analyses of GP, DFs, dough, and bread

##### Determination of moisture content

The moisture contents of the untreated GP and MW‐treated GP were determined using the thermal drying method in a drying oven (99200–3, Stanhope‐Seta) at 105°C for 12 h by following Wang et al. (2007) procedure.

##### Microstructure analysis of DFs

The microstructures of the untreated and MW‐treated SDF and IDF samples were examined using a scanning electron microscope (JSM IT‐700HR, JEOL). Each sample was coated with gold with a sputter coater (Structure Probe) for 4 min before being scanned and photographed at 450× magnification. An accelerating potential of 5 kV was used during micrography.

##### Hydration properties of DFs

The solubility index (SI), water‐holding capacity (WHC), swelling power (SP), and sediment volume fraction (SVF) of the SDF and IDF samples were measured at 25°C by using the centrifugal technique with some modifications (Robertson et al., 2000). Accordingly, 0.5 g of each sample was dispersed in 20 mL of deionized water and vortexed for 60 s. Afterward, the dispersed samples were incubated at 25°C for 12 h. The incubated dispersion was centrifuged (Hettich EBA 200) at 4000 × *g* for 20 min. The supernatant was poured into a tared beaker and put in an oven at 120°C to evaporate the moisture. The SI, WHC, SP, and SVF results of the SDF and IDF samples were calculated according to the Robertson et al. (2000) study.

##### Rheological characteristics of doughs

The mixing profiles of the control and enriched doughs were determined using a farinograph (827504, Brabender) according to AACC Method 54–21 (AACC, 2000). Water absorption (%), mixing tolerance index (MTI) (B.U.), dough development time (DDT) (min), and dough stability (min) were measured using a farinograph test. The elastic properties of the control and enriched dough samples were also measured using an extensograph (860001, Brabender) following AACC Method 54–10 (AACC, 2000). Resistance to extension (B.U.), extensibility (mm), proportional number, and energy (cm^2^) were reported from the extensograph test.

##### Physical properties of breads

Specific volume (cm^3^/g), weight loss (%), and crust color difference (∆E*) of the breads were measured and the weight loss was calculated according to Equation (1):

(1) $$\text{Weight loss}\;\left(\%\right)=\frac{W_{i}-W_{f}}{W_{i}}\times 100$$

where *W*_*i*_ and *W*_*f*_ represent the weight of the dough and the bread, respectively.

According to AACC Method 10–05, the volumes (cm^3^) of breads were measured by seed (rape) displacement and the specific volumes were calculated using Equation (2):

(2) $$\text{Specific volume}\;\left({\mathrm{cm}}^{3}/g\right)=\frac{\text{Volume}}{\text{Weight}}$$

Color values of the bread samples were detected in a Color Flex colorimeter (Hunter). The instrument was calibrated with standard tiles (black and white), and the color values of the white tile were taken as a reference (*L*ₒ = 94.41, *a*ₒ = −0.98, and *b*ₒ = −0.35). The color difference (∆E*) for the crust was calculated using Equation (3):

(3) $$∆E*=\sqrt{{\left(Lₒ-L\right)}^{2}+{\left(aₒ-a\right)}^{2}+{\left(bₒ-b\right)}^{2}}$$

where *L* is the lightness (brightness), *a* is redness: green to red, and *b* stands for yellowness: blue to yellow.

##### Textural properties of breads

After removing the crusts, the bread samples were cut to 2 × 2 × 2 cm in size. Hardness (g), cohesiveness, springiness, and chewiness (g) were measured from the midsection of the bread samples by using a Texture Analyzer (TA‐XT Plus C, Stable Micro Systems) with a 36‐mm‐diameter cylindrical probe, 50% compression, and a test speed of 2.0 mm s^−1^. Measurements were reported with an average of three replicates. Chewiness values (g) were obtained from Equation (4):

(4) $$\text{Chewiness}\;\left(g\right)=\left(\text{hardness}\times \text{cohesiveness}\times \text{springiness}\right)$$

#### 2.2.4 Statistical analysis

The statistical analysis was performed using SPSS 23 (IBM). All measurements were performed in triplicate. The control, U‐SDF, U‐IDF, MW‐SDF, and MW‐IDF groups were compared using an analysis of variance (ANOVA) by means of Tukey's HSD test. The results are presented as mean ± SD (*n* = 3). The significance level was *p* < 0.05.

#### 2.2.5 Limitation of the study

The results may have differed from samples processed in an industrial‐type (915 MHz) MW oven, because MW‐treated samples were treated in a home‐type (2450 MHz) MW oven. In addition, AOAC 991.43 procedure was followed in DF analysis; thus, low‐molecular‐weight SDF content could not be measured in SDF samples.

## 3 RESULTS AND DISCUSSION

### 3.1 Microstructures of DFs

**FIGURE 1:**
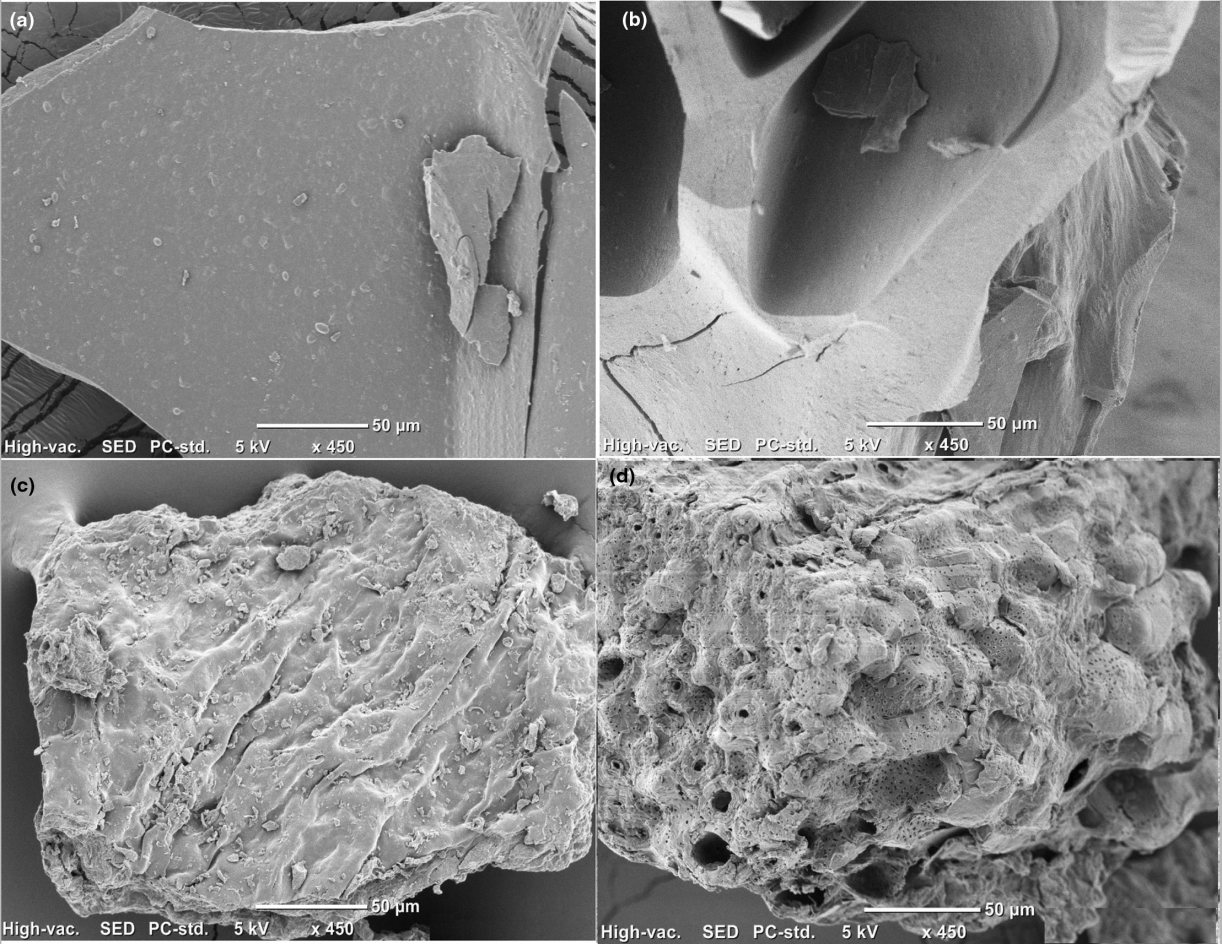
Microstructures of dietary fibers: (a) untreated soluble dietary fiber isolate, (b) microwave‐treated soluble dietary fiber isolate, (c) untreated insoluble dietary fiber isolate, and (d) microwave treated insoluble dietary fiber isolate.

The microstructures of the untreated and MW‐treated SDF and IDF are illustrated in Figure 1. The isolation step provided clear images because the proteins and carbohydrates within the fibers were hydrolyzed, and the utilized chemicals washed out all fragments and compounds other than DF from the structure surfaces. Even the U‐SDF displayed a smooth structure with sharp edges (Figure 1a). After MW treatment (MW‐SDF), this clean‐cut structure was corrugated, and honeycomb‐like structures developed (Figure 1b), which can be attributed to the rapid heating mechanism of MW and the pressure gradient from fast steam generation. Similarly, Gan et al. (2020) stated that the crystal structure of grapefruit peel SDF was damaged considerably after MW application. As compared to SDF, rugged and nubbly morphology was observed in IDF. There were also wrinkles on surface with prevalent irregularly shaped fragments (Figure 1c). MW treatment generated a large number of holes that formed from the inside to the outside (Figure 1d). This porous structure might have formed due to the absorption of microwave energy by the cells, thereby causing cellular rupture (Wei et al., 2019). A loose morphology might improve the hydration properties of DF fractions.

### 3.2 Hydrating properties of DFs

Hydration properties might affect the rheological, textural, and multiple other physical properties of the products. The SI, WHC, SP, and SVF values of the DF samples are summarized in Table 1. Due to SI being an indicator of SDF's presence in the samples, the values of SI were found higher in the SDF samples compared to the IDF samples. This result is consistent with the output of a study by Oh et al. (2014) that claims that a decrease in SDF ratio causes lower SI scores. MW treatment increased SI values in both SDF and IDF samples. This improvement may have resulted from the development of porosity, capillary attraction, and hydrophilia as an effect of the MW energy, as also reported by Oliveira et al. (2020). In addition, Bader Ul Ain et al. (2019) stated that high temperatures break the glycosidic bonds of polysaccharides, which may lead to the release of oligosaccharides and increase solubility of DF. Regarding the other hydration indicators of DF, the WHC and SP of the IDF samples were found to be higher than those of the SDF group. This may be due to higher water affinity due to the indented porous structure of IDF as reported by Chen et al. (2019). Similar results were obtained in a study by Daou and Zhang (2011) on hemicellulose and lignin; MW application improved WHC and SP scores. Braşoveanu and Nemţanu (2014) explained this mechanism via the fact that MW rearranged the intermolecular structure; therefore, water permeation eased up, improving the hydration abilities of the DF. Cell ruptures and loose IDF matrices due to pressure gradients arising from the internal heating mechanism of MW could have also contributed to this consequence. The microstructure images of DF fractions provide valuable information about their hydrating properties. Protrusions observed in microstructures, wrinkles, holes, and cavities on the surface increase surface area and porosity. All of these structures promote the water‐holding and swelling capacity of the material.

**TABLE 1:** Hydrating properties of dietary fiber isolates.

DF samples	SI (%)	WHC (g/g)	SP (g/g)	SVF (%)
U‐SDF	45.89 ± 0.26^b^	3.90 ± 0.12^d^	10.88 ± 0.17^d^	9.85 ± 0.31^c^
MW‐SDF	56.83 ± 0.29^a^	7.56 ± 0.35^c^	15.93 ± 0.05^c^	5.49 ± 0.15^d^
U‐IDF	29.05 ± 0.12^d^	13.09 ± 0.11^b^	29.92 ± 0.11^b^	42.71 ± 0.24^a^
MW‐IDF	38.03 ± 0.07^c^	14.93 ± 0.21^a^	37.02 ± 0.02^a^	37.92 ± 0.12^b^

*Note*: Superscript letters ‘^a,b,c,d^’ show statistically significant differences in columns (*p* < .05).

Abbreviations: MW‐IDF, microwave‐treated insoluble dietary fiber isolate; MW‐SDF, microwave‐treated soluble dietary fiber isolate; SI, solubility index; SP, swelling power; SVF, sediment volume fraction; U‐IDF, untreated insoluble dietary fiber isolate; U‐SDF, untreated soluble dietary fiber isolate; WHC, water‐holding capacity.

SVF represents the insoluble fraction rate in the compound. For this reason, SVF values were found higher in IDFs, as expected. MW application reduced SVF scores in both DF groups. This reduction might be dependent on decreasing particle size due to the molecular collision generated by MW (Jiang et al., 2020).

### 3.3 Rheological characteristics

#### 3.3.1 Farinograph measurements

Rheological properties are parameters that give information about dough structure and directly affect the quality of final product (i.e., bread). Rheological properties are presented as farinograph and extensograph measurements. The farinograph measurements present the parameters used to determine the kneading properties of the dough and provide information about the breading properties of the material used. The farinograph parameters of the control and the enriched doughs are summarized in Table 2. The water absorption rate changed significantly depending on whether the bread was enriched with SDF or IDF (*p* < .05). These results could be explained as effects of the hydroxyl groups in the hydrocolloids, as these functional groups allow more water interaction through hydrogen bonding and might increase water absorption. The highest absorption was observed in the IDF‐enriched samples. Daou and Zhang (2011) reached similar results and provided an explanation for the presence of hemicellulose and lignin in IDF, which exhibit higher water affinity. It is also known that the effects of IDF, which include reducing transit time through the gut and increasing fecal bulk, are based on its high water absorption ability (Oh et al., 2014). A similar result was found in the Chouaibi et al. (2019) study on insoluble tomato fiber‐enriched dough. Conversely, SDF decreased the water absorption of the dough (Table 2) in relation to the low SP and WHC values of SDF. Likewise, Brennan et al. (2004) found that inulin, an SDF source, inhibited water absorption by 23%, and the swelling index of the dough decreased compared to control pasta.

**TABLE 2:** Farinograph parameters of doughs.

Dough samples	Water absorption (%)	Mixing tolerance index (BU)	Dough development time (min)	Dough stability (min)
Control	60.77 ± 2.10^c^	31.48 ± 0.90^e^	1.63 ± 0.33^e^	2.20 ± 0.13^e^
Control+U‐SDF	51.20 ± 1.75^e^	40.18 ± 0.32^d^	2.25 ± 0.10^d^	3.16 ± 0.15^d^
Control+MW‐SDF	55.32 ± 1.14^d^	46.65 ± 1.10^c^	3.51 ± 0.03^c^	3.88 ± 0.08^c^
Control+U‐IDF	72.10 ± 1.49^b^	68.60 ± 4.70^b^	4.05 ± 0.05^b^	4.62 ± 0.42^b^
Control+MW‐IDF	78.97 ± 1.19^a^	77.98 ± 1.89^a^	4.85 ± 0.12^a^	5.30 ± 0.22^a^

*Note*: Superscript letters ‘^a,b,c,d,e^’ show statistically significant differences in columns (*p* < .05).

Abbreviations: Control, 100% wheat flour dough (no fiber fraction added); Control+MW‐SDF, microwave‐treated soluble dietary fiber isolate‐enriched dough; Control+U‐IDF, untreated insoluble dietary fiber isolate‐enriched dough; Control+MW‐IDF, microwave‐treated insoluble dietary fiber isolate‐enriched dough; Control+U‐SDF, untreated soluble dietary fiber isolate‐enriched dough.

Regarding the MW‐treated DF, the water absorption capacity rates of the dough with the MW‐treated DF increased. This finding is consistent with the WHC of the MW‐treated DFs. Absorption rates could be affected by the surface area expansion of the MW‐treated DFs due to particle size reduction. The developed holes and grooves in the microstructures of the MW‐treated DFs (Figure 1b,d) also support this result. Jiang et al. (2020) clarified that the concurrent expansion of the surface area and reducing particle size is due to MW‐induced molecular collisions.

DF addition influences dough structure via different mechanisms: (1) DF dilutes and thereby weakens the gluten network; (2) DF molecules hinder the formation of bonds (steric effect) in the gluten network; (3) DF competes with gluten and starch molecules for water (Morris & Morris, 2012). These three phenomena inhibit gluten network development. Higher MTI values suggest that the dough became weaker due to delayed gluten matrix development; thus, the dough became less resistant to mechanical force during mixing. As shown in Table 2, MTI values increased in the enriched dough considerably due to the dilution of gluten protein with the DFs, which may be explained by the interaction between fiber and gluten influencing the consistency and mixing properties of the dough.

A high DDT value indicates that more time is required to develop the dough. Hydrocolloids such as SDF and IDF cause an increase in DDT, as DF competes with flour for water in the period of development and interferes with the starch–gluten matrix. Therefore, the water absorption of dough is slower, and the time needed for development is longer. The increase in DDT demonstrates a delay in the hydration and formation of the gluten network. In this period, the composite dough starts to get stronger (Chouaibi et al., 2019). DDT was increased to a different extent by each hydrocolloid type. In this study, IDF considerably increased DDT, WHC, and MTI, whereas these parameters were increased to a lesser extent by SDF (Table 2). The results regarding the delay in dough development coincide with the findings of Ortiz de Erive et al. (2020) for soluble oat fiber‐enriched doughs and those of Chouaibi et al. (2019) for insoluble tomato fiber‐enriched wheat doughs.

Stability is an indication of dough strength, with longer stability times suggesting stronger doughs. According to the data shown in Table 2, the incorporation of either SDF or IDF extended the stability time of dough. These results also show higher interactions among water, DF, and gluten. Moreover, these were expected results, as MTI was found to be higher than the control. Longer stability times coincided with the degree of dough softening (MTI) as well. The increased stability time of SDF in this study is consistent with that observed in inulin‐enriched dough (Wang et al., 2002); IDF's stability time is in agreement with that of tomato insoluble fiber‐fortified dough (Chouaibi et al., 2019).

#### 3.3.2 Extensograph measurements

The extensograph gives information about the balance of the elastic and viscous properties of dough. The extensograph parameters (resistance to extension, extensibility, and energy) of the dough fortified with DFs are presented in Table 3. The elasticity of the dough was reduced with the incorporation of any of the hydrocolloids tested, indicating that viscoelasticity decreases with the addition of DF as well. The greatest reduction in the extensograph parameters was observed after IDF enrichment. The gluten protein in flour fortifies the dough's extensograph properties. DFs compete for water with the macromolecules present in dough (e.g., starch, gluten) (Oh et al., 2014). IDFs, which have high water absorption capacity, seize the water that would otherwise be absorbed by gluten. The partial hydration of gluten weakens covalent bonds (disulfide bonds); thus, the extensograph characteristics of the dough were negatively affected. Moreover, the fact that the water absorption rates of the doughs with IDF were higher than those with SDF added, as indicated in Table 2, confirms this possible mechanism for lower extensograph parameters. Effects on particle size and surface characteristics can be observed after MW application (Jiang et al., 2020). Surface area extension and an increase in the pore volume of DFs developed with MW treatment might have caused the extensograph values of the enriched doughs to change.

**TABLE 3:** Extensograph parameters of doughs.

Dough samples	Resistance to extension (BU)	Extensibility (mm)	Energy (cm^2^)
Control	789.27 ± 8.64^a^	137.22 ± 4.36^a^	88.48 ± 1.93^a^
Control+U‐SDF	617.90 ± 8.10^c^	112.17 ± 6.08^b^	63.62 ± 2.09^c^
Control+MW‐SDF	672.65 ± 7.52^b^	86.43 ± 3.58^c^	71.37 ± 2.61^b^
Control+U‐IDF	404.58 ± 11.45^e^	75.78 ± 2.05^d^	51.56 ± 1.21^e^
Control+MW‐IDF	571.88 ± 9.06^d^	62.85 ± 2.05^e^	56.93 ± 0.81^d^

*Note*: Superscript letters ‘^a,b,c,d,e^’ show statistically significant differences in columns (*p* < .05).

Abbreviations: Control, 100% wheat flour dough (no fiber fraction added); Control+MW‐SDF, microwave‐treated soluble dietary fiber isolate‐enriched dough; Control+U‐IDF, untreated insoluble dietary fiber isolate‐enriched dough; Control+MW‐IDF, microwave‐treated insoluble dietary fiber isolate‐enriched dough; Control+U‐SDF, untreated soluble dietary fiber isolate‐enriched dough.

The enrichment of DF weakened the dough, which caused a decrease in its resistance to extension. The resistance to the extension values presented in Table 3 are consistent with this finding and were reduced by the addition of DF. This can be attributed to the interaction between proteins and polysaccharides suggested by Jones and Erlander (1967). IDF decreased resistance more than SDF; this indicates that IDF weakened the dough structure more than SDF by interfering more with gluten and starch due to its complex molecular configuration.

Extensibility indicates dough handling characteristics. By adding water to the bread formula, glutenin and gliadin proteins in flour begin to hydrate, interact, and develop cross‐linked bonds that can hold the carbon dioxide produced by the yeast. This forms the extensibility of the dough. The influence of DF incorporation on dough extensibility was evident; according to the data in Table 3, DF enrichment reduced the extensibility values of the doughs. Likewise, a decrease in dough extensibility after the addition of DF from oat whole meal and carob was found in a study by Miś et al. (2012) as well. In addition, the extensibility of the dough decreased with increasing bran amounts. This may be related to the dilution of gluten proteins by the addition of DF (Sudha et al., 2007). The lowest extensibility was recorded after the addition of IDF, just like the rest of the extensograph parameters. Extensibility is an indicator of the swelling ability of the gluten matrix. IDF competes for water more than SDF, as it needs more water to swell and it holds more water than SDF. The high SP and WHC values of IDF presented in Table 1 support this mechanism. Therefore, IDF prevents the gluten from swelling by absorbing the water in the environment. This reduces the extensibility property of the dough to a greater extent than SDF. The extensibility values of the dough with MW‐treated DF decreased considerably; this can be attributed to the MW treatment improving the WHC of the SDF as well as the SP of both the IDF and SDF. Moreover, the microstructures of the MW‐treated DFs also support the potential variations in water–fiber interactions.

Significant energy reduction was observed after replacing flour (Table 3). The changing trend of energy values was consistent with varying values of resistance to extension. The lowest values were observed for the IDF‐incorporated doughs. Compared to the IDF‐enriched dough, the energy values of the dough with SDF were found to be closer to those of the control dough.

### 3.4 Physical properties of breads

#### 3.4.1 Weight loss

Certain quality characteristics of the bread samples are summarized in Table 4. Weight loss and specific volume results are interrelated. It was expected that specific volume would decrease with a decrease in weight loss, as breads with lower weight loss rates are heavy and cannot rise during baking. In the bread cooling period, moisture is released from bread at a faster rate; however, water evaporation was limited in the DF‐enriched breads due to the hydrophilic characteristic of DF. According to the results in Table 4, the lowest weight loss rates were found in IDF‐enriched breads, and this can be attributed to the higher hydration ability of IDF. In a study by Mancebo et al. (2018), SDF did not change moisture content in cookies, whereas IDF increased it significantly. IDF caused steric hindrance and prevented the formation of cross‐linked network structures in dough. Therefore, IDF retains and does not release water, unlike SDF.

**TABLE 4:** Physical properties of breads.

Bread samples	Weight loss (%)	Specific volume (cm^3^/g)	Crust color difference (∆E*)
Control	10.07 ± 0.23^a^	5.63 ± 0.36^a^	69.14 ± 1.51^a^
Control+U‐SDF	8.25 ± 0.17^b^	3.95 ± 0.36^b^	58.37 ± 1.36^b^
Control+MW‐SDF	8.38 ± 0.25^b^	3.64 ± 0.18^cb^	51.15 ± 0.98^c^
Control+U‐IDF	5.85 ± 0.20^c^	3.09 ± 0.33^d^	44.14 ± 1.03^d^
Control+MW‐IDF	5.68 ± 0.22^c^	3.14 ± 0.08^d^	42.69 ± 0.87^d^

*Note*: Superscript letters ‘^a,b,c,d^’ show statistically significant differences in columns (*p* < .05).

Abbreviations: Control, 100% wheat flour bread (no fiber fraction added); Control+MW‐SDF, microwave‐treated soluble dietary fiber isolate‐enriched bread; Control+U‐IDF, untreated insoluble dietary fiber isolate‐enriched bread; Control+MW‐IDF, microwave‐treated insoluble dietary fiber isolate‐enriched bread; Control+U‐SDF, untreated soluble dietary fiber isolate‐enriched bread.

#### 3.4.2 Specific volume

After it is added into dough, DF absorbs free water in the media and leaves too little water for gluten to develop a network. This leads to a reduction in the gas‐holding capacity of the dough. As a result, under‐risen dough and breads with small loaf volumes are obtained. In addition, DF prevents the development of a strong network (formation of disulfide bonds) by entering between gluten molecules. This can weaken the dough and decrease the specific volume by reducing the amount of CO_2_ captured (Arı Akın et al., 2021; Ni et al., 2020). This mechanism is supported by the data in Table 4. The specific volumes of the DF‐enriched bread were found to be lower than the control, and IDF‐added samples further influenced the final volume of the breads. The effective mechanism might be that SDF only retains water by forming hydrogen bonds, whereas IDF entraps water within its intercellular spaces and porous structure (Mudgil, 2017). Therefore, IDF holds more water, increasing the weight of the final IDF‐enriched product and decreasing the rising ability of the dough more than SDF. Consequently, heavier products with lower volumes have low specific volumes.

#### 3.4.3 Crust color difference

The crust color difference values of the bread samples are presented in Table 4. In the DF‐added products, the crust color changed mainly depending on caramelization and Maillard reactions (Arı Akın et al., 2021). Since SDF and IDF do not contain amino acids and only consist of nonstarch polysaccharides, the formation of Maillard and caramelization reaction products may have decreased during baking as compared to control bread (100% flour). This also reduced the color difference.

### 3.5 Textural properties of breads

Significant changes in textural properties were observed for all DF‐enriched breads (*p* < .05), as shown in Table 5. The hardness values of all of the enriched breads were greater than the control, but unlike IDF, the addition of SDF made up the difference and brought the results closer to that of the control. This effect may be associated with the strong compactness of IDF particles in doughs due to their geometry, whereas SDF loses its original shape by dissolving in water. Mancebo et al. (2018) also explained the changes in the hardness of cookies after SDF and IDF addition with the same reasons. Similarly, Xu et al. (2021) observed that the bread hardness increased up to fivefold with DF supplementation. In breads prepared using okra flour, which has high SDF content, this rate was limited to threefold. On the other hand, hardness values decreased, approaching that of the control sample; this may be due to MW treatment destroying the compactness of IDF. In terms of SDF, MW treatment also improved the solubility of SDF particles in water by increasing the SI value, thereby contributing to the decrease of the hardness value by reducing the amount of suspension in the formulation.

**TABLE 5:** Textural properties of breads.

Bread samples	Hardness (g)	Cohesiveness	Springiness	Chewiness (g)
Control	288.18 ± 9.33^e^	0.87 ± 0.02^a^	1.24 ± 0.07^a^	310.19 ± 29.95^a^
Control+U‐SDF	384.71 ± 9.91^c^	0.72 ± 0.04^b^	0.88 ± 0.03^b^	244.88 ± 17.43^b^
Control+MW‐SDF	318.61 ± 2.62^d^	0.80 ± 0.02^a^	0.94 ± 0.03^b^	239.31 ± 2.94^b^
Control+U‐IDF	460.29 ± 5.86^a^	0.56 ± 0.03^d^	0.73 ± 0.02^c^	186.08 ± 5.41^c^
Control+MW‐IDF	410.43 ± 7.19^b^	0.64 ± 0.02^c^	0.71 ± 0.02^c^	187.37 ± 0.84^c^

*Note*: Superscript letters ‘^a,b,c,d,e^’ show statistically significant differences in columns (*p* < .05).

Abbreviations: Control, 100% wheat flour bread (no fiber fraction added); Control+MW‐SDF, microwave‐treated soluble dietary fiber isolate‐enriched bread; Control+U‐IDF, untreated insoluble dietary fiber isolate‐enriched bread; Control+MW‐IDF, microwave‐treated insoluble dietary fiber isolate‐enriched bread; Control+U‐SDF, untreated soluble dietary fiber isolate‐enriched bread.

Cohesiveness describes how well a product retains its form between the first and second chew. Springiness is the extent of recovery after the removal of force. Increased hardness is related to decreased cohesiveness and springiness. The results of this study are also consistent with this finding.

As presented in Table 5, the DF composite breads are less cohesive with lower springiness values than the control breads. IDF decreased the cohesiveness and springiness values of the bread more than SDF. By adding MW‐treated DF, cohesiveness and springiness values approached those of the control. Compared to flour particles, the interlocking polymeric structures of DFs with a high absorption capacity can inhibit the development of the gluten network, the ability to retain gases, and the formation of an elastic, less cohesive, and springier crumb structure. However, the clear and more homogeneous distribution of cell particles and the ability of SDFs to quickly disperse in water have provided a more cohesive crumb structure with higher springiness values than IDF. The results and this mechanism are also supported by Xu et al. (2021). Moreover, because MW treatment reduces particle size, it can be hypothesized that MW‐treated DFs are better dispersed in the dough than in untreated DFs, and therefore, cohesiveness scores increase because they create a more cohesive and homogeneous structure.

Chewiness is like a summary of textural properties, as it expresses a combination of hardness, cohesiveness, and springiness; this property reflects the energy required to masticate food to a state ready for swallowing. SDF and IDF substitution markedly decreased bread chewiness. In fact, chewiness values are highly dependent on springiness; therefore, they were reduced (186.08–310.19 g) by decreasing springiness. The changes in springiness directly caused the changes in crumb elasticity. Similarly, the change in the chewiness of the breads, according to the changing trend of springiness, coincides with the Onyango et al. (2010) study for sorghum bread with α‐amylase and cassava starch. This modification of chewiness is based on increased DF content, its high water absorption abilities, and dilution of the gluten matrix.

## 4 CONCLUSION

In this study, MW treatment was utilized to modify the IDF and SDF of grapes, and the structural and functional properties of IDF and SDF and their effects on bread quality were investigated. The results of the SDF‐enriched samples were found to be closer to those of the control breads' values than those of the IDF‐enriched ones. MW treatment developed holes and grooves in the microstructures of DF and improved hydration properties compared to untreated DF. This improvement was also consistent with an increase in the SI, WHC, and SP of both DF fractions. IDF‐enriched breads had the highest farinograph measurements and the lowest scores in extensograph properties compared to the SDF‐enriched and control doughs. The weight loss, specific volume, crust color difference, cohesiveness, springiness, and chewiness of the IDF‐enriched breads were found to be the lowest. The addition of SDF provided farinographic, extensographic, textural, and other physical properties closer to the control than IDF fortification. MW treatment improved SDF's structure by helping it obtain water absorption rates, resistance to extension, energy, hardness, cohesiveness, and springiness scores closer to the control. It also modified IDF's properties to attain better resistance to extension, energy, hardness, and cohesiveness values in the IDF‐enriched breads compared to the control results. The importance of this study is as follows: (1) a 2‐min MW application changed the functional and technological properties of DFs; (2) in the enrichment of bread, it is recommended to use SDFs as a flour substitute at a certain rate (5% flour basis) rather than IDF; (3) MW treatment is more effective on SDFs than IDFs, and it is more effective in achieving results closer to control breads; (4) this study can serve as an example for food waste reduction, and these findings can provide a framework for further studies. For further research, the best bread formulations can be developed for SDF and IDF.

## AUTHOR CONTRIBUTIONS

**Duygu Baskaya‐Sezer:** Conceptualization (lead); data curation (lead); formal analysis (lead); investigation (lead); methodology (lead); resources (lead); software (lead); supervision (lead); validation (lead); visualization (lead); writing – original draft (lead); writing – review and editing (lead).

## FUNDING INFORMATION

This research did not receive any specific grant from funding agencies in the public, commercial, or not‐for‐profit sectors.

## CONFLICT OF INTEREST STATEMENT

The author has no relationship or conflict of interest with any person or company.

## ETHICS STATEMENT

The study does not need ethical approval. The material was not reproduced using other sources in the study.

## Data Availability

The data that support the findings of this study are available on request from the corresponding author.
